# Higher carbohydrate quality, but not quantity, is associated with lower lung cancer risk in a population-based prospective cohort study

**DOI:** 10.3389/fnut.2026.1857838

**Published:** 2026-07-23

**Authors:** Xiaoxiang Zhu, Ming Tang, Yuanbo Xue, Qian Zhan, Yi Xiao, Yuxiang Luo, Linguo Shen, Wenkui Yu

**Affiliations:** 1 Department of Critical Care Medicine, Nanjing Drum Tower Hospital, The Affiliated Hospital of Nanjing University Medical School, Nanjing, Jiangsu, China; 2 Department of Emergency Medicine, The Affiliated Suqian Hospital of Xuzhou Medical University, Suqian, Jiangsu, China; 3 Center of Urology and Nephrology, The Second Affiliated Hospital of Chongqing Medical University, Chongqing, China; 4 Department of Critical Care Medicine, Nanjing Drum Tower Hospital, Clinical College of Nanjing University of Chinese Medicine, Nanjing, Jiangsu, China; 5 Department of Respiratory Medicine, The Second Affiliated Hospital of Chongqing Medical University, Chongqing, China; 6 Center for Lipid Research, The Second Affiliated Hospital, Chongqing Medical University, Chongqing, China; 7 Department of Cardiology, Cardiovascular Institute, Thoraxcenter, Erasmus University Medical Center, Rotterdam, Netherlands

**Keywords:** carbohydrate quality index, cox regression analysis, low-carbohydrate diet, lung cancer, PLCO trial

## Abstract

**Background:**

The role of diet in lung cancer is increasingly recognized, but it remains unclear whether carbohydrate quality or carbohydrate quantity is more relevant to risk. We therefore examined the associations of the carbohydrate quality index (CQI) and low-carbohydrate diet score (LCD) with incident lung cancer in a population-based prospective cohort.

**Methods:**

We included 101,755 participants from the Prostate, Lung, Colorectal, and Ovarian (PLCO) Cancer Screening Trial. CQI and LCD were used to assess carbohydrate quality and quantity, respectively. Cox proportional hazards models were used to estimate hazard ratios (HRs) and 95% confidence intervals (CIs) for lung cancer across quartiles of CQI and LCD. Restricted cubic spline, subgroup, and sensitivity analyses were conducted to assess dose–response relationships and the robustness of the findings.

**Results:**

During 897,809.4 person-years of follow-up, 1,706 incident lung cancer cases were identified. Higher CQI was significantly associated with a lower risk of lung cancer after multivariable adjustment. In the fully adjusted model, compared with the lowest quartile, the HRs (95% CIs) for lung cancer were 0.82 (0.72, 0.93), 0.78 (0.68, 0.89), and 0.73 (0.64, 0.84) for the second, third, and fourth quartiles of CQI, respectively (*P* for trend <0.001). By contrast, LCD was not significantly associated with lung cancer risk after multivariable adjustment, with corresponding HRs (95% CIs) of 0.93 (0.81, 1.07), 1.02 (0.89, 1.17), and 1.09 (0.95, 1.25) across increasing quartiles (*P* for trend = 0.117). Restricted cubic spline analyses showed a significant non-linear inverse association for CQI (*P* for nonlinearity = 0.006), but not for LCD (*P* for nonlinearity = 0.150). The inverse association for CQI remained robust in multiple sensitivity analyses, including further adjustment for LCD and alternative smoking exposure models.

**Conclusion:**

Higher carbohydrate quality, but not carbohydrate quantity, was associated with a lower risk of lung cancer, suggesting a greater relevance of carbohydrate quality to lung cancer risk.

## Introduction

Lung cancer remains one of the most common malignancies and the leading cause of cancer-related death worldwide, posing a substantial public health burden (1). Although tobacco exposure is the predominant risk factor, approximately 25% of lung cancer cases occur in never-smokers (2, 3), indicating that lung carcinogenesis cannot be fully explained by smoking alone. Increasing evidence suggests that other modifiable factors, particularly diet, may also contribute to lung cancer development (4–6). Identifying dietary exposures associated with lung cancer risk may therefore provide additional opportunities for prevention.

Among dietary exposures potentially related to lung carcinogenesis, carbohydrate-related factors warrant particular attention. Although carbohydrates are a major source of dietary energy, their metabolic effects may vary substantially according to nutritional quality (7). In this context, carbohydrate quality may be especially relevant to lung cancer risk, as higher-quality carbohydrate intake is associated with more favorable glycemic and metabolic profiles and greater consumption of whole grains and fiber-rich foods, which together may influence lung carcinogenesis (8, 9).

Existing studies on carbohydrates and lung cancer have mainly examined individual carbohydrate-related components rather than overall carbohydrate quality (10–12). Evaluating isolated components may not adequately capture the multidimensional nutritional and metabolic properties of carbohydrate intake. The carbohydrate quality index (CQI), which integrates dietary fiber, glycemic index, the ratio of whole grains to total grains, and the ratio of solid to total carbohydrates, provides a more comprehensive assessment of carbohydrate quality (13). Recent studies have suggested that carbohydrate quality may be more relevant than carbohydrate quantity for certain cancer outcomes (14), but whether carbohydrate quality and quantity differ in their associations with lung cancer risk remains unclear.

Therefore, in this prospective analysis of the Prostate, Lung, Colorectal, and Ovarian (PLCO) Cancer Screening Trial, we primarily investigated the association between CQI and lung cancer risk. We additionally evaluated the low-carbohydrate diet score (LCD) as a secondary comparison to explore whether carbohydrate quantity was associated with lung cancer risk.

## Materials and methods

### Study design and population

This prospective cohort study was conducted using data from the PLCO Cancer Screening Trial, a large multicenter randomized trial sponsored by the National Cancer Institute (NCI) in the United States. The PLCO trial enrolled 154,887 men and women aged 55 to 74 years from 10 screening centers between 1993 and 2001. Participants were randomly assigned to either the intervention arm or the control arm. Baseline demographic and clinical information were collected using the Baseline Questionnaire (BQ), whereas habitual dietary intake during the previous year was assessed using the Diet History Questionnaire (DHQ), a validated food frequency questionnaire (15). Nutrient and food group intakes were derived from DHQ responses with the aid of DietCalc software. In the present study, follow-up time was calculated from the date of DHQ completion until the first occurrence of lung cancer diagnosis, death, loss to follow-up, or December 31, 2009, whichever came first. The study timeline and follow-up scheme are illustrated in Supplementary Figure S1.

**Figure 1:**
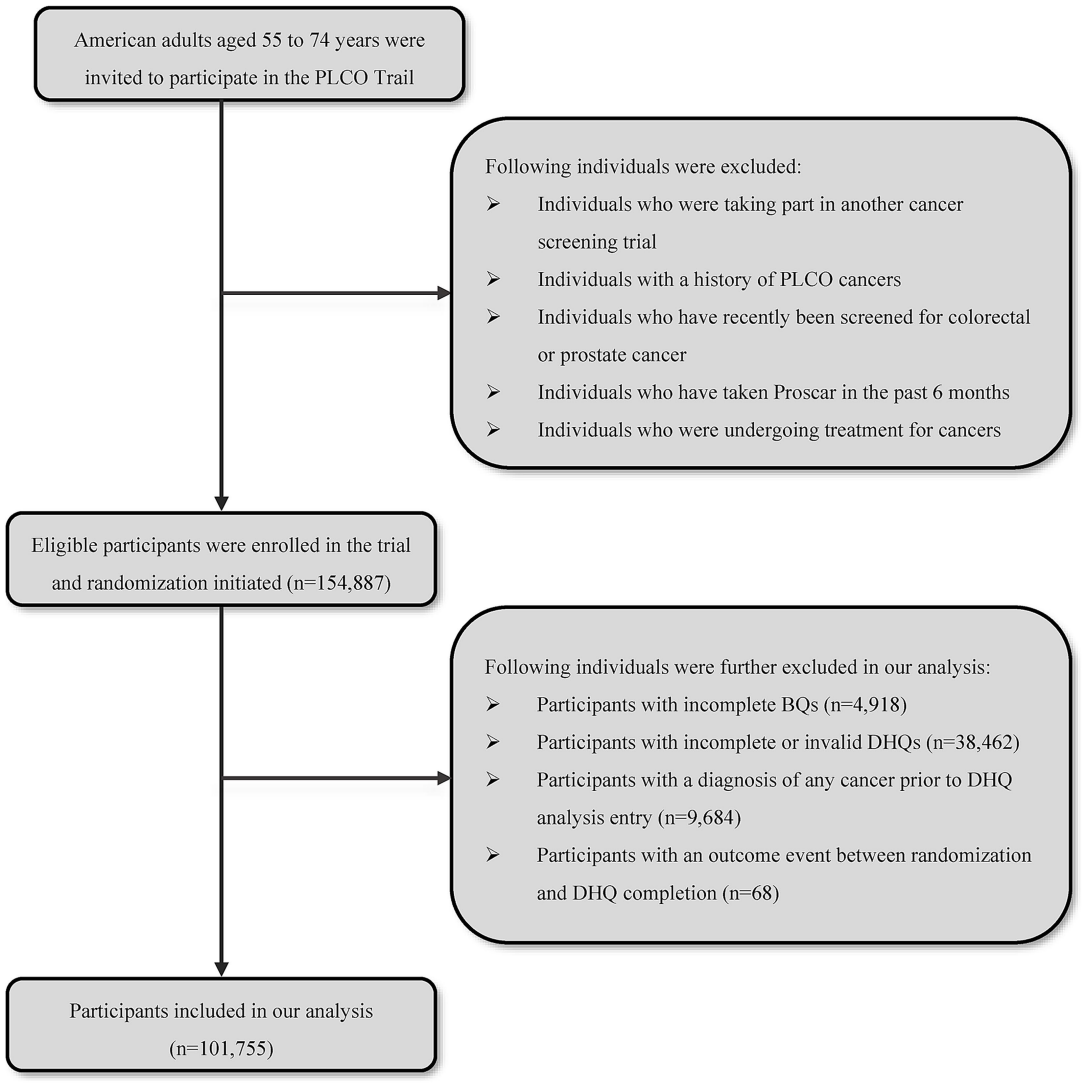
The flow chart of identifying participants included in this study.

Participants were excluded if they did not complete the BQ (*n* = 4,918), did not complete the DHQ or submitted an invalid DHQ (*n* = 38,462), had a history of any cancer before DHQ completion (*n* = 9,684), or were diagnosed with lung cancer between DHQ entry and DHQ completion (*n* = 68). An invalid DHQ was defined as a questionnaire with at least eight missing frequency responses. The detailed inclusion and exclusion process is shown in Figure 1. After these exclusions, 101,755 participants remained in the final analytic cohort. This study was conducted using PLCO data under application number PLCO-1886.

### Assessment of carbohydrate quality and quantity

The carbohydrate quality index (CQI) and low-carbohydrate diet score (LCD) were calculated to assess carbohydrate quality and quantity, respectively (7, 16). CQI was constructed to reflect overall carbohydrate quality across four domains: dietary fiber intake, glycemic index (GI), the ratio of whole grains to total grains, and the ratio of solid carbohydrates to total carbohydrates. For each component, participants were classified into quintiles. Higher quintiles of dietary fiber, whole grain-to-total grain ratio, and solid-to-total carbohydrate ratio were assigned higher scores, whereas GI was scored in reverse order. Component scores were summed to generate a total CQI ranging from 4 to 20, with higher values indicating better carbohydrate quality. CQI was calculated according to a previously published method in which dietary fiber intake, glycemic index, the ratio of whole grains to total grains, and the ratio of solid to total carbohydrates were given equal weight. We retained this established approach to ensure comparability with prior studies, as no validated weighting scheme currently exists for lung cancer-related outcomes. In this calculation, liquid carbohydrates were estimated from sugar-sweetened beverages and alcohol, and total grains were defined as the sum of whole grains, refined grains, and related grain products.

LCD was used to assess the quantitative restriction of carbohydrate intake. Briefly, the percentages of total energy derived from carbohydrate, fat, and protein were calculated for each participant and ranked across 11 equal-sized strata. Carbohydrate intake was inversely scored, such that lower carbohydrate intake received higher scores, whereas fat and protein were positively scored. The three component scores were summed to yield an LCD ranging from 0 to 30. Higher LCD values represented a more pronounced low-carbohydrate dietary pattern, characterized by relatively lower carbohydrate intake and higher fat and protein intake. Detailed criteria for the calculation of CQI and LCD are provided in Supplementary Table S1.

### Ascertainment of lung cancer

Incident lung cancer was the primary outcome of this study. Cases were identified during follow-up through annual study update forms, study screenings, self-reports, family reports, and data linkages with cancer registries and the National Death Index. Additional follow-up by telephone or email was undertaken when participants did not respond to the annual forms. For all reported lung cancer cases, medical and pathological records were reviewed to confirm the diagnosis and obtain further information on the malignancy. Histologic information was coded according to the International Classification of Diseases for Oncology, 2nd Edition (ICD-O-2) morphology.

### Assessment of covariates

Potential confounders were selected according to previously published literature and the availability of relevant variables in the PLCO dataset (17, 18). The Baseline Questionnaire (BQ) provided information on sex, race/ethnicity, education level, smoking status, history of hypertension, history of diabetes, family history of lung cancer, and body mass index (BMI), which was calculated as weight in kilograms divided by height in meters squared. For analysis, race/ethnicity was categorized as White and Non-white to avoid sparse data in smaller racial/ethnic subgroups. Age, total dietary energy intake, and drinking status were obtained from the Diet History Questionnaire (DHQ). Physical activity level, defined as the weekly duration of moderate-to-vigorous activity, was ascertained from a supplemental questionnaire.

### Statistical analysis

Missing covariate data were addressed according to the degree of missingness. For variables with less than 5% missing data, categorical variables were imputed with the mode and continuous variables with the median (19, 20). Specifically, mode imputation was used for race/ethnicity, education level, smoking status, drinking status, history of hypertension, history of diabetes, and family history of lung cancer, whereas BMI was imputed with the median. Physical activity level, which had 25.6% missingness, was imputed using multiple imputation by chained equations with predictive mean matching (25 imputations and 5 iterations), with all variables in the analytic dataset included in the imputation model, The imputed values were then combined to generate a completed analytic dataset for the main analyses (21). This hybrid strategy was adopted because missingness was minimal for most covariates, while the variable with substantial missingness was addressed using a multiple imputation framework. For the sensitivity analyses using alternative smoking exposure indicators, smoking status was first imputed using the modal category (never-smokers). Never-smokers were assigned a value of 0 for both cigarettes smoked per day and pack-years, whereas missing values among current/former smokers were imputed using the mode for cigarettes smoked per day and the median for pack-years. Detailed distributions of variables before and after imputation are shown in Supplementary Table S2. All subsequent analyses were based on the imputed dataset.

Associations of CQI and LCD with lung cancer risk were assessed using Cox proportional hazards regression models, with follow-up time as the underlying time scale. The proportional hazards assumption was evaluated using Schoenfeld residuals, and no substantial violation was observed. Both scores were analyzed in quartiles, using the lowest quartile as the reference category. An unadjusted model was initially constructed, followed by Model 1 adjusted for age, sex, race/ethnicity, and education level. Model 2 was additionally adjusted for body mass index, smoking status, drinking status, history of hypertension, history of diabetes, family history of lung cancer, physical activity level, and dietary energy intake. Additional smoking exposure indicators were evaluated in sensitivity analyses rather than entered simultaneously into the primary model because of potential multicollinearity among smoking-related variables and to further assess residual confounding by smoking exposure (22, 23).

Linear trends across quartiles were tested by assigning the median value of each quartile to participants in the corresponding category and entering this variable into the model as a continuous term. Potential dose–response relationships between carbohydrate-related scores and lung cancer risk were further explored using restricted cubic spline analyses within the Cox proportional hazards framework, with adjustment for the same covariates as in Model 2. For each score, restricted cubic spline models with 3 to 7 knots were compared, and the optimal model was selected according to the minimum Akaike information criterion. Based on this criterion, a 3-knot model was selected for CQI, with knots placed at the 10th, 50th, and 90th percentiles, whereas a 5-knot model was selected for LCD, with knots placed at the 5th, 27.5th, 50th, 72.5th, and 95th percentiles of the LCD distribution. Nonlinearity was assessed using analysis of variance.

Prespecified subgroup analyses were performed according to sex (male vs. female), age (<65 vs. ≥65 years), BMI (<25 vs. ≥25 kg/m^2^), smoking status (never vs. current/former), drinking status (never vs. current/former), history of hypertension (no vs. yes), history of diabetes (no vs. yes), family history of lung cancer (no vs. yes/possible), and dietary energy intake (< median vs. ≥ median). Within each subgroup, multivariable Cox proportional hazards models were fitted to estimate hazard ratios (HRs) and 95% confidence intervals (CIs) for the comparison of the highest versus lowest quartile of CQI (Q4 vs. Q1). All covariates included in the main model were adjusted for, except the corresponding stratification variable. Interaction was assessed using likelihood ratio tests by comparing models with and without the CQI-by-subgroup interaction term among participants in the lowest and highest quartiles of CQI. A two-sided *P* for interaction <0.05 was considered statistically significant.

To assess the robustness of the primary findings, several sensitivity analyses were performed. Specifically, we repeated the analyses after excluding participants with missing covariate data, those with a family history of lung cancer, those with a history of hypertension or diabetes, and those with extreme dietary energy intake (<800 kcal/day or >4,200 kcal/day for men, and <600 kcal/day or >3,500 kcal/day for women). We also excluded lung cancer cases diagnosed within the first 2 years of follow-up to minimize potential reverse causation (24). Sensitivity analyses were also conducted to further assess smoking-related confounding. Specifically, smoking status in the fully adjusted model was replaced by cigarettes smoked per day or pack-years, respectively. We also fitted an additional model including both smoking status and pack-years simultaneously. Because smoking-related variables are correlated, this model was interpreted as a sensitivity analysis rather than as the primary model. To examine whether the observed association was influenced by carbohydrate quantity, LCD was further included in Model 2 of the CQI analysis. All statistical analyses were conducted using R version 4.5.3, and a two-sided *p* value <0.05 was considered statistically significant.

## Results

### Baseline characteristics

A total of 101,755 participants were included in the present analysis. The mean (standard deviation [SD]) CQI was 12.0 (3.1), and the mean LCD was 15.0 (7.1). Baseline characteristics according to quartiles of CQI are presented in Table 1, and those according to quartiles of LCD are shown in Supplementary Table S3. Participants in the highest quartile of CQI were generally older, more likely to be White and postgraduate educated, had lower BMI, lower smoking exposure, lower glycemic index, higher fiber intake, higher whole-grain and solid carbohydrate intake, lower liquid carbohydrate intake, greater physical activity, and higher dietary energy intake than those in the lowest quartile. In contrast, participants in the highest quartile of LCD tended to be younger, more likely to be male, have higher BMI, greater smoking exposure, higher prevalence of diabetes, lower physical activity, and higher dietary energy intake.

**Table 1:** Baseline characteristics of study population according to quartiles of carbohydrate quality index (CQI)^a^.

Characteristics	Overall	Quartiles of carbohydrate quality index (CQI)			
		Quartile 1 (≤10)	Quartile 2 (11–12)	Quartile 3 (13–14)	Quartile 4 (≥15)
Number of participants	101,755	32,436	23,621	22,652	23,046
Carbohydrate Quality Index (CQI)	12.0 ± 3.1	8.4 ± 1.6	11.5 ± 0.5	13.5 ± 0.5	16.1 ± 1.2
Age (years)	65.5 ± 5.7	64.6 ± 5.6	65.5 ± 5.7	65.9 ± 5.7	66.5 ± 5.7
Gender					
Male	49,496 (48.6%)	15,370 (47.4%)	11,564 (49.0%)	11,232 (49.6%)	11,330 (49.2%)
Female	52,259 (51.4%)	17,066 (52.6%)	12,057 (51.0%)	11,420 (50.4%)	11,716 (50.8%)
Race/ethnicity					
White	94,066 (92.4%)	28,717 (88.5%)	21,901 (92.7%)	21,329 (94.2%)	22,119 (96.0%)
Non-white	7,689 (7.6%)	3,719 (11.5%)	1720 (7.3%)	1,323 (5.8%)	927 (4.0%)
Education level					
Some college or less	64,953 (63.8%)	21,807 (67.2%)	15,193 (64.3%)	14,092 (62.2%)	13,861 (60.1%)
College graduate	17,848 (17.5%)	5,343 (16.5%)	4,139 (17.5%)	4,070 (18.0%)	4,296 (18.6%)
Postgraduate	18,954 (18.6%)	5,286 (16.3%)	4,289 (18.2%)	4,490 (19.8%)	4,889 (21.2%)
Body mass index (kg/m^2^)	27.2 ± 4.8	27.7 ± 5.0	27.3 ± 4.8	27.0 ± 4.7	26.6 ± 4.6
Smoking status					
Never	48,580 (47.7%)	14,376 (44.3%)	11,271 (47.7%)	11,119 (49.1%)	11,814 (51.3%)
Current/former	53,175 (52.3%)	18,060 (55.7%)	12,350 (52.3%)	11,533 (50.9%)	11,232 (48.7%)
Cigarettes smoked per day when smoked					
Never-smoker	48,580 (47.7%)	14,376 (44.3%)	11,271 (47.7%)	11,119 (49.1%)	11,814 (51.3%)
1–10	13,596 (13.4%)	4,315 (13.3%)	3,127 (13.2%)	2,983 (13.2%)	3,171 (13.8%)
11–20	19,727 (19.4%)	6,682 (20.6%)	4,609 (19.5%)	4,339 (19.2%)	4,097 (17.8%)
≥21	19,852 (19.5%)	7,063 (21.8%)	4,614 (19.5%)	4,211 (18.6%)	3,964 (17.2%)
Packs smoked per day* years	17.7 ± 26.6	20.3 ± 28.4	17.7 ± 26.6	16.5 ± 25.5	15.1 ± 24.6
Drinking status					
Never	10,116 (9.9%)	3,101 (9.6%)	2,237 (9.5%)	2,240 (9.9%)	2,538 (11.0%)
Current/former	91,639 (90.1%)	29,335 (90.4%)	21,384 (90.5%)	20,412 (90.1%)	20,508 (89.0%)
History of hypertension					
No	68,707 (67.5%)	20,916 (64.5%)	15,899 (67.3%)	15,573 (68.7%)	16,319 (70.8%)
Yes	33,048 (32.5%)	11,520 (35.5%)	7,722 (32.7%)	7,079 (31.3%)	6,727 (29.2%)
History of diabetes					
No	94,949 (93.3%)	30,150 (93.0%)	21,960 (93.0%)	21,241 (93.8%)	21,598 (93.7%)
Yes	6,806 (6.7%)	2,286 (7.0%)	1,661 (7.0%)	1,411 (6.2%)	1,448 (6.3%)
Family history of lung cancer					
No	88,738 (87.2%)	28,082 (86.6%)	20,605 (87.2%)	19,798 (87.4%)	20,253 (87.9%)
Yes/possible	13,017 (12.8%)	4,354 (13.4%)	3,016 (12.8%)	2,854 (12.6%)	2,793 (12.1%)
Physical activity level (min/week)	122.9 ± 108.8	106.4 ± 101.3	120.4 ± 107.3	129.2 ± 110.2	142.4 ± 115.2
Dietary factors					
Energy intake from diet (kcal/day)	1738.6 ± 736.4	1519.5 ± 654.2	1704.8 ± 729.0	1843.0 ± 746.8	1979.1 ± 749.7
Glycemic index	53.6 ± 3.3	55.3 ± 3.0	53.8 ± 3.1	52.9 ± 3.0	51.4 ± 2.8
Fiber intake (g/day)	18.0 ± 8.5	12.7 ± 5.3	16.8 ± 6.9	20.0 ± 7.8	24.8 ± 8.9
Solid carbohydrates intake (g/day)	221.5 ± 91.5	187.7 ± 83.0	214.8 ± 88.9	236.8 ± 90.4	260.9 ± 87.8
Liquid carbohydrates intake (g/day)	409.8 ± 476.4	574.3 ± 582.6	416.8 ± 467.0	344.0 ± 401.2	235.9 ± 264.2
Whole grains intake (g/day)	61.1 ± 59.9	32.0 ± 36.5	55.7 ± 50.5	73.1 ± 59.6	96.0 ± 72.8
Refined grains intake (g/day)	103.6 ± 93.6	91.5 ± 86.4	103.6 ± 95.5	111.7 ± 96.8	112.5 ± 96.4

a Values are means (standard deviation) for continuous variables and percentages for categorical variables.

### Associations of CQI and LCD with lung cancer risk

During 897,809.4 person-years of follow-up, 1,706 incident lung cancer cases were documented. As shown in Table 2, higher CQI was significantly associated with a lower risk of lung cancer. In the fully adjusted model, compared with participants in the lowest quartile, the hazard ratios (HRs) and 95% confidence intervals (CIs) were 0.82 (0.72, 0.93) for quartile 2, 0.78 (0.68, 0.89) for quartile 3, and 0.73 (0.64, 0.84) for quartile 4 (*P* for trend <0.001). Similar inverse associations were observed in the unadjusted and minimally adjusted models.

**Table 2:** Association of CQI and LCD score with the risk of lung cancer.

Quartiles	No. of participants	No. of cases	Person-years	Hazard ratio (95% confidence interval)		
				Unadjusted	Model 1^a^	Model 2^b^
Carbohydrate Quality Index (CQI)						
Quartile 1 (≤10)	32,436	631	285003.9	1.00 (reference)	1.00 (reference)	1.00 (reference)
Quartile 2 (11–12)	23,621	383	208534.2	0.83 (0.73, 0.94)	0.78 (0.69, 0.89)	0.82 (0.72, 0.93)
Quartile 3 (13–14)	22,652	353	200473.1	0.79 (0.70, 0.91)	0.74 (0.65, 0.84)	0.78 (0.68, 0.89)
Quartile 4 (≥15)	23,046	339	203798.3	0.75 (0.66, 0.86)	0.68 (0.59, 0.78)	0.73 (0.64, 0.84)
*P* for trend				<0.001	<0.001	<0.001
Low-carbohydrate Diet Score (LCD)						
Quartile 1 (≤10)	28,881	428	258145.1	1.00 (reference)	1.00 (reference)	1.00 (reference)
Quartile 2 (11–15)	24,757	372	219732.0	1.02 (0.89, 1.18)	1.03 (0.90, 1.19)	0.93 (0.81, 1.07)
Quartile 3 (16–20)	23,776	419	209338.9	1.21 (1.06, 1.39)	1.23 (1.07, 1.41)	1.02 (0.89, 1.17)
Quartile 4 (≥21)	24,341	487	210593.5	1.40 (1.23, 1.60)	1.47 (1.29, 1.68)	1.09 (0.95, 1.25)
*P* for trend				<0.001	<0.001	0.117

a Adjusted for age (continuous), gender (male, female), race/ethnicity (white, non-white) and education level (some college or less, college graduate and postgraduate).

b Adjusted for model 1 plus body mass index (kg/m^2^, continuous), smoking status (never, current/former), drinking status (never, current/former), history of hypertension (no, yes), history of diabetes (no, yes), family history of lung cancer (no, yes/possible), physical activity level (min/week, continuous), and energy intake from diet (kcal/day, continuous).

By contrast, LCD was not significantly associated with lung cancer risk after multivariable adjustment. Although higher LCD was associated with an increased risk of lung cancer in the unadjusted and minimally adjusted models, the association was attenuated and no longer statistically significant after full adjustment. In Model 2, the HRs (95% CIs) were 0.93 (0.81, 1.07) for quartile 2, 1.02 (0.89, 1.17) for quartile 3, and 1.09 (0.95, 1.25) for quartile 4, with no significant linear trend (*P* for trend = 0.117).

### Dose–response analyses

**Figure 2:**
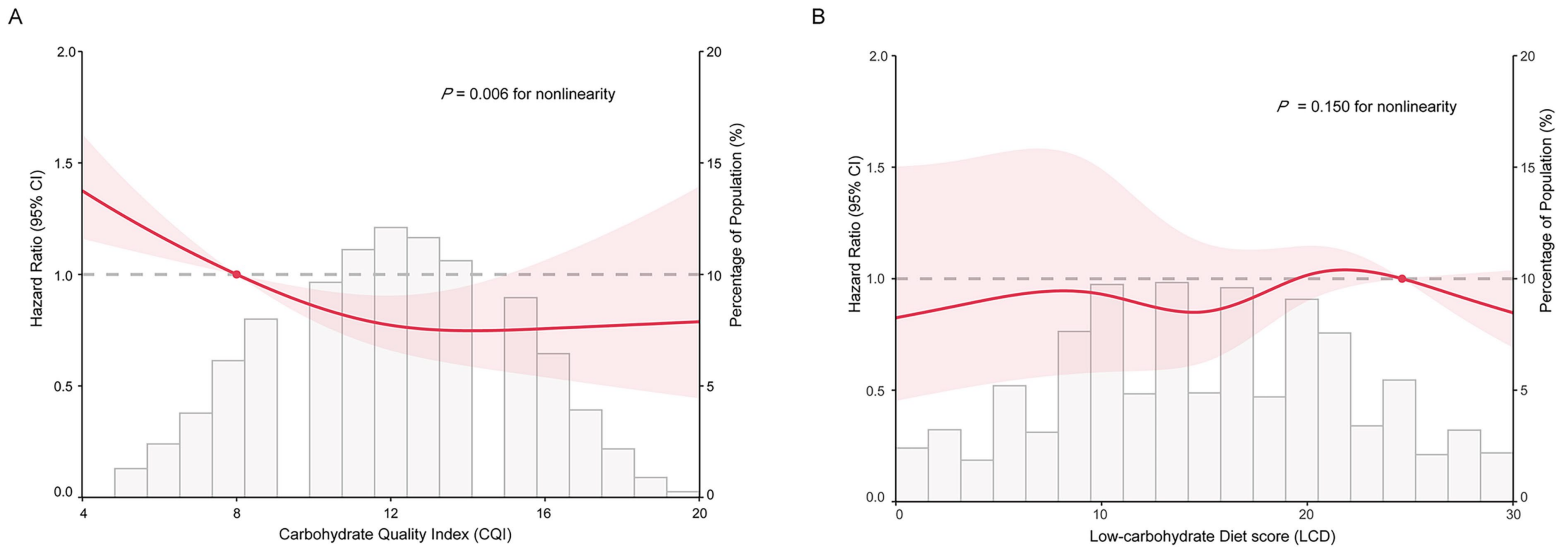
Dose–response associations of CQI **(A)** and LCD score **(B)** with lung cancer risk.

Restricted cubic spline analyses further characterized the dose–response relationships between carbohydrate-related scores and lung cancer risk. As shown in Figure 2A, CQI displayed a significant non-linear inverse association with lung cancer risk (*P* for nonlinearity = 0.006), with risk decreasing more noticeably across the lower-to-moderate range of CQI and then tending to flatten at higher values, suggesting a possible plateau rather than a distinct threshold. In contrast, Figure 2B showed no significant non-linear association between LCD and lung cancer risk (*P* for nonlinearity = 0.150). The spline curve remained close to HR = 1.0 across most of the exposure range, with no clear threshold or plateau effect.

### Subgroup analyses

**Figure 3:**
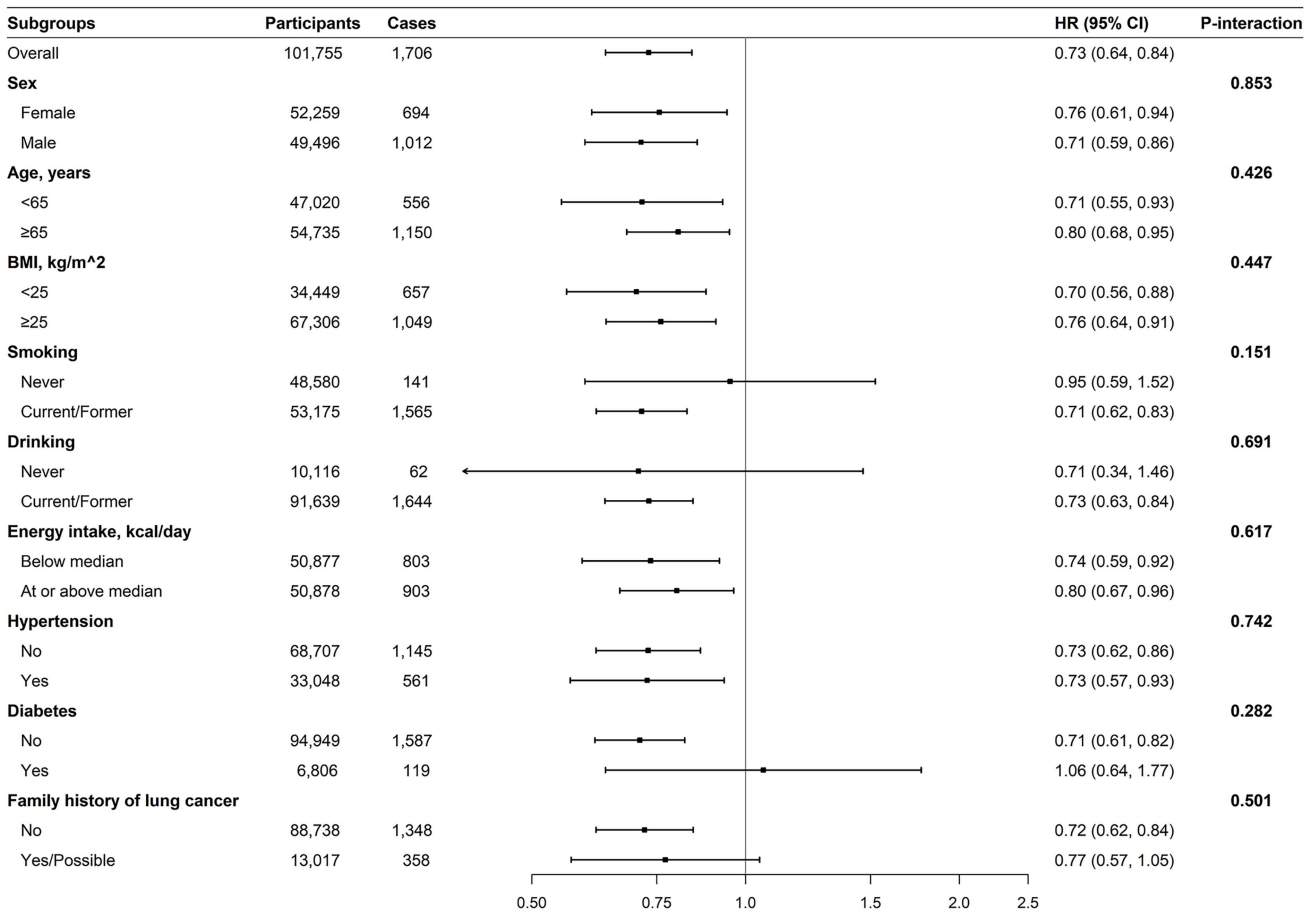
Subgroup analyses on the association between CQI and lung cancer risk.

In prespecified subgroup analyses, the inverse association between CQI and lung cancer risk was generally consistent across strata defined by sex, age, BMI, smoking status, drinking status, dietary energy intake, history of hypertension, history of diabetes, and family history of lung cancer (Figure 3). The association appeared more evident among current/former smokers and among participants without diabetes, whereas no clear association was observed among never-smokers or participants with diabetes. However, no statistically significant interaction was observed for any subgroup variable (all *P* for interaction >0.05).

### Sensitivity analyses

The inverse association between CQI and lung cancer risk remained robust across multiple sensitivity analyses (Table 3), including exclusion of participants with missing covariate data, a family history of lung cancer, a history of hypertension or diabetes, extreme dietary energy intake, and lung cancer cases diagnosed within the first 2 years of follow-up. To further characterize smoking-related differences in lung cancer occurrence, incidence rates according to smoking status and CQI quartiles are presented in Supplementary Table S4.

**Table 3:** Sensitivity analyses on the association of carbohydrate quality index (CQI) with the risk of lung cancer.

Categories	No. of participants	No. of cases	HR _Quartile 4 vs. Quartile 1_ (95% confidence interval)^a^	*P* for trend
Primary analysis	101,755	1706	0.73 (0.64, 0.84)	<0.001
Excluded cases observed within the first 2 years of follow-up	101,447	1,398	0.72 (0.62, 0.84)	<0.001
Excluded participants with history of hypertension^b^	68,707	1,145	0.73 (0.62, 0.86)	<0.001
Excluded participants with history of diabetes^c^	94,949	1,587	0.71 (0.61, 0.82)	<0.001
Excluded participants with family history of lung cancer^d^	88,738	1,348	0.72 (0.62, 0.84)	<0.001
Exclude participants with missing data	71,772	729	0.78 (0.63, 0.97)	0.011
Excluded participants with extreme dietary energy intake^e^	98,459	1,642	0.74 (0.64, 0.85)	<0.001
Adjusted for cigarettes/day instead of smoking status	101,755	1706	0.78 (0.68, 0.90)	<0.001
Adjusted for pack-years instead of smoking status	101,755	1706	0.85 (0.74, 0.98)	0.015
Adjusted for smoking status and pack-years simultaneously	101,755	1706	0.86 (0.75, 0.99)	0.026
Further adjusted for Low-carbohydrate Diet score (LCD)	101,755	1706	0.74 (0.65, 0.86)	<0.001

a Adjusted for age (continuous), gender (male, female), race/ethnicity (white, non-white), education level (some college or less, college graduate and postgraduate), body mass index (kg/m^2^, continuous), smoking status (never, current/former), drinking status (never, current/former), history of hypertension (no, yes), history of diabetes (no, yes), family history of lung cancer (no, yes/possible), physical activity level (min/week, continuous), and energy intake from diet (kcal/day, continuous).

b Hazard ratio was not adjusted for history of hypertension.

c Hazard ratio was not adjusted for history of diabetes.

d Hazard ratio was not adjusted for family history of lung cancer.

e Extreme dietary energy intake was classified as <800 kcal/day or > 4,200 kcal/day for men, and < 600 kcal/day or > 3,500 kcal/day for women.

Additional sensitivity analyses using alternative smoking exposure indicators yielded directionally consistent results. The association remained statistically significant when smoking status was replaced by cigarettes smoked per day [HR 0.78, 95% CI 0.68–0.90] or by pack-years [HR 0.85, 95% CI 0.74–0.98]. In the additional model including both smoking status and pack-years, the association between CQI and lung cancer risk remained statistically significant [HR 0.86, 95% CI 0.75–0.99]. Further adjustment for LCD also did not materially alter the primary association [HR 0.74, 95% CI 0.65–0.86].

## Discussion

In this population-based prospective cohort study, we found that higher carbohydrate quality, as reflected by CQI, was associated with a lower risk of lung cancer, whereas carbohydrate quantity, assessed by LCD, was not associated with lung cancer risk after multivariable adjustment. Participants in the highest quartile of CQI had a 27% lower risk of lung cancer than those in the lowest quartile, and this inverse association was further supported by dose–response analyses. By contrast, LCD showed no significant association with lung cancer risk in the fully adjusted model. Notably, the primary association for CQI remained robust in multiple sensitivity analyses, including further adjustment for LCD and alternative parameterizations of smoking exposure, although the effect estimate was attenuated when smoking status was replaced by pack-years. The spline analyses provided additional support for the differential roles of carbohydrate quality and quantity. For CQI, the inverse association was non-linear, with the largest reduction in risk observed across the lower-to-moderate range and a flatter curve at higher values, suggesting that the association may weaken beyond a certain level rather than continue linearly. By contrast, LCD showed no clear threshold or plateau pattern, further supporting the absence of a meaningful dose–response association between carbohydrate quantity and lung cancer risk in this cohort. Taken together, these findings suggest that carbohydrate quality may be more relevant than carbohydrate quantity in relation to lung cancer risk in this population.

Several biologically plausible mechanisms may underlie the observed association between carbohydrate quality and lung cancer risk, although these pathways were not directly examined in the present study. First, higher-quality carbohydrates are less likely to induce exaggerated postprandial glycemic excursions and hyperinsulinemia, which may reduce activation of the insulin/IGF-1 axis, a pathway implicated in cell proliferation, survival signaling, and tumor development (25). In this context, a lower glycemic index and a greater proportion of fiber-rich and whole-grain carbohydrates may provide a more favorable metabolic profile than carbohydrate quantity alone. Second, carbohydrate quality may influence oxidative stress. Previous studies have shown that higher dietary glycemic index or glycemic load is associated with greater lipid peroxidation and other markers of oxidative stress (26), whereas better carbohydrate quality appears to be more strongly linked to lower oxidative burden than total carbohydrate intake itself. Third, these metabolic effects may translate into differences in chronic inflammation. Evidence from respiratory research suggests that specific carbohydrate components, such as dietary fiber and free sugars, are differentially associated with chronic lung disease and lung function, partly through the regulation of low-grade inflammation (27), while experimental studies indicate that highly digestible carbohydrate-rich diets can aggravate airway inflammation, mucus hypersecretion, inflammatory cell infiltration, and TH2/TH17-skewed immune responses (28). Finally, higher carbohydrate quality may also be associated with gut microbial features related to butyrate precursor metabolism and immune regulation (8, 29, 30). However, because biomarkers of insulin signaling, oxidative stress, inflammation, and gut microbiota were not measured in the present study, these mechanisms should be interpreted as hypothesis-generating explanations based on prior literature rather than pathways directly supported by our data.

Previous epidemiologic studies on carbohydrate-related exposures and lung cancer have mainly focused on individual dimensions of carbohydrate quality, with mixed findings across different carbohydrate-related components. Prospective studies of glycemic index (GI) and glycemic load (GL) have reported mixed results: the Southern Community Cohort Study observed no independent association of either GI or GL with lung cancer risk (31), whereas a more recent PLCO-based analysis found a positive association for GI but an inverse association for GL (12). In addition, higher dietary fiber and whole-grain intake have been associated with lower lung cancer risk in some prospective studies (5, 10, 11). Recent UK Biobank data further suggested that dietary fiber and non-free sugars were inversely associated with lung cancer, whereas free sugars and sucrose were positively associated (32). However, these studies assessed only isolated dimensions of carbohydrate quality and therefore could not adequately capture its multidimensional nature or distinguish the effects of specific components from those of overall carbohydrate quality. To date, only one previous study has directly examined carbohydrate quality indices in relation to lung cancer (33). That hospital-based case–control study from Iran reported inverse associations for both CQI and LCDS, but its retrospective design, hospital-based control selection, and relatively small sample size limit the strength and generalizability of the findings. Against this background, our population-based prospective analysis extends the literature by simultaneously evaluating CQI and LCD in a large cohort, providing a more robust assessment of carbohydrate quality versus quantity in relation to lung cancer risk.

In our study, higher LCD appeared to be associated with an increased risk of lung cancer in the unadjusted and minimally adjusted models, but this association disappeared after full adjustment. This pattern suggests that the crude association of LCD with lung cancer risk was likely confounded by lifestyle and metabolic factors closely related to low-carbohydrate dietary patterns, including smoking exposure, BMI, diabetes, and total energy intake. It should also be noted that LCD reflects a broader macronutrient distribution pattern characterized by relatively lower carbohydrate intake together with higher fat and protein intake, rather than pure carbohydrate quantity in isolation. Accordingly, the null association observed for LCD should be interpreted as applying to this low-carbohydrate dietary pattern score rather than to all possible aspects of carbohydrate quantity. The inverse association between CQI and lung cancer risk remained materially unchanged after further adjustment for LCD, indicating that this association was not solely explained by carbohydrate quantity. The persistence of this association after adjustment for multiple lifestyle and metabolic covariates also argues against the possibility that CQI was merely acting as a proxy for a generally healthier lifestyle. In sensitivity analyses using alternative smoking exposure indicators, the estimate for CQI remained stable when smoking status was replaced by cigarettes smoked per day. When smoking status was replaced by pack-years, the effect estimate for CQI was attenuated from 0.73 to 0.85. This attenuation indicates that cumulative smoking exposure accounted for a meaningful part of the observed association. In an additional model including both smoking status and pack-years, the association remained statistically significant, further suggesting that smoking exposure may partly, but not fully, explain the inverse association between higher CQI and lung cancer risk. Nevertheless, given the central role of smoking in lung cancer etiology, residual confounding by smoking cannot be completely ruled out.

In subgroup analyses, the inverse association between CQI and lung cancer risk was generally consistent across prespecified strata, and no statistically significant effect modification was observed. Although the association appeared somewhat stronger among current/former smokers and participants without diabetes, these subgroup-specific findings should be interpreted cautiously because the interaction tests were not significant. The lack of a clear association among never-smokers and participants with diabetes could partly reflect the smaller number of lung cancer cases in these strata and the resulting limited statistical power, rather than indicating true effect heterogeneity.

Several strengths of this study should be acknowledged. First, the prospective cohort design, large sample size, and prospective ascertainment of lung cancer outcomes in a well-characterized cohort reduced the likelihood of recall bias and provided sufficient statistical power to evaluate the associations of carbohydrate-related indices with lung cancer risk. Second, by using CQI as an integrated measure of carbohydrate quality, our study moved beyond isolated carbohydrate components and provided a more comprehensive assessment of the nutritional and metabolic characteristics of carbohydrate intake. Third, we simultaneously evaluated CQI and LCD and performed multiple sensitivity analyses, including alternative parameterizations of smoking exposure and further adjustment for LCD, which strengthened the robustness and interpretability of our findings.

This study also has several limitations. First, dietary intake was assessed using a baseline food frequency questionnaire, which is subject to measurement error and may not fully reflect changes in diet over time. Nevertheless, the DHQ used in PLCO was a validated instrument designed to capture usual dietary intake over the preceding year, and food frequency questionnaires remain a practical and widely accepted approach for assessing long-term dietary exposure in large prospective cohort studies (34). In addition, although we used a pragmatic missing-data strategy based on the extent of missingness, alternative approaches such as a unified multiple imputation framework could be considered in future studies. Second, as with all observational studies, residual confounding cannot be completely excluded, particularly for smoking-related factors. Although alternative models using cigarettes smoked per day and pack-years yielded directionally consistent results, the attenuation observed after pack-year adjustment suggests that some residual confounding by cumulative smoking exposure may remain. Moreover, the observed association may still partly reflect broader healthy lifestyle or dietary patterns not fully captured in our models. Relatedly, because biological markers of insulin metabolism, oxidative stress, inflammation, and the gut microbiota were not available, the mechanistic explanations discussed above should be interpreted as hypothesis-generating rather than directly demonstrated in this study. Third, because the PLCO cohort consisted mainly of middle-aged and older US adults, the generalizability of our findings to younger populations or other ethnic and geographic groups may be limited. Finally, the interpretation of the two carbohydrate-related indices also requires caution. LCD reflects carbohydrate quantity only indirectly and also captures a broader low-carbohydrate dietary pattern characterized by higher fat and protein intake. CQI, in turn, assigns equal weight to its four components, although their relative importance for lung cancer risk may not be identical. In addition, the solid-to-total carbohydrate ratio may partly reflect beverage-related behaviors, including alcohol and other liquid carbohydrate sources, rather than carbohydrate quality alone, and CQI may still overlap with broader overall diet quality. These issues should be considered when interpreting the observed associations.

In conclusion, our findings suggest that higher carbohydrate quality, as reflected by CQI, was associated with a lower risk of lung cancer, whereas LCD-defined low-carbohydrate dietary pattern was not independently associated with lung cancer risk in this cohort. Given the observational design, these findings should be interpreted as associations and do not establish a causal relationship between carbohydrate quality and lung cancer risk. Further prospective studies in other populations, as well as mechanistic studies, are needed to confirm these findings.

## Data Availability

The data analyzed in this study is subject to the following licenses/restrictions: the data can be accessed upon reasonable request and with permission from the National Cancer Institute. Requests to access these datasets should be directed to https://cdas.cancer.gov/learn/plco/trial-summary/.

## Supplementary material

SUPPLEMENTARY FIGURE S1 The timeline and follow-up scheme of this study.
