# Supplementary material for: Higher carbohydrate quality, but not quantity, is associated with lower lung cancer risk in a population-based prospective cohort study
Source: Front Nutr. 2026 Jul 23;13:1857838. doi: 10.3389/fnut.2026.1857838 (PMC13441921; doi:10.3389/fnut.2026.1857838)
Supplement: Supplementary file 1 [file Table_1.DOCX]

**Table S1.** Criteria for determining CQI and LCD score.

| **Components** | **Range (points)** | **Minimum index** | **Maximum index** |
| --- | --- | --- | --- |
| **Carbohydrate quality index (CQI)** |  |  |  |
| 1. Dietary fiber intake (g/d) | 1-5 | Minimum intake (first quintile) | Maximum intake (fifth quintile) |
| 2. Glycemic index | 1-5 | Maximum index (fifth quintile) | Minimum index (first quintile) |
| 3. Ratio of whole-grain: total-grain carbohydrates | 1-5 | Minimum value of this ratio (first quintile) | Maximum value of this ratio (fifth quintile) |
| 4. Ratio of solid carbohydrates: (solid carbohydrates+ liquid carbohydrates) | 1-5 | Minimum value of this ratio (first quintile) | Maximum value of this ratio (fifth quintile) |
| **Total index (range)** | **4-20** | | |
| **Low-carbohydrate diet score (LCD)** |  |  |  |
| 1. Carbohydrate intake (% E) **^a^** | 0-10 | Maximum intake (first 1/11) | Minimum intake (last 1/11) |
| 2. Protein intake (% E) | 0-10 | Minimum intake (last 1/11) | Maximum intake (first 1/11) |
| 3. Fat intake (% E) | 0-10 | Minimum intake (last 1/11) | Maximum intake (first 1/11) |
| **Total score (range)** | **0-30** | | |

**^a^** %E, the percentage of total energy intake.
