# Supplementary material for: Higher carbohydrate quality, but not quantity, is associated with lower lung cancer risk in a population-based prospective cohort study
Source: Front Nutr. 2026 Jul 23;13:1857838. doi: 10.3389/fnut.2026.1857838 (PMC13441921; doi:10.3389/fnut.2026.1857838)
Supplement: Supplementary file 2 [file Table_2.DOCX]

**Table S2.** Distribution of covariates with missing data before and after imputation **^a^**

| **Variable** | **Before imputation** | **After imputation** | **Number (%) with missing data** |
| --- | --- | --- | --- |
| **Race/ethnicity** |  |  | 37 (0.04%) |
| White | 94029 (92.44%) | 94066 (92.44%) |  |
| Non-White | 7689 (7.56%) | 7689 (7.56%) |  |
| **Education level** |  |  | 204(0.20%) |
| Some college or less | 64749 (63.8%) | 64953 (63.8%) |  |
| College graduate | 17848 (17.6%) | 17848 (17.5%) |  |
| Postgraduate | 18954 (18.7%) | 18954 (18.6%) |  |
| **Body mass index (kg/m^2^)** | 27.2±4.8 | 27.2±4.8 | 1348 (1.32%) |
| **Smoking status** |  |  | 20 (0.02%) |
| Never | 48560 (47.7%) | 48580 (47.7%) |  |
| Current/ Former | 53175 (52.3%) | 53175 (52.3%) |  |
| **Cigarettes smoked per day when smoked** |  |  | 125 (0.12%) |
| Never-smoker | 48560 (47.8%) | 48580 (47.7%) |  |
| 1-10 | 13596 (13.4%) | 13596 (13.4%) |  |
| 11-20 | 19622 (19.3%) | 19727 (19.4%) |  |
| ≥21 | 19852 (19.5%) | 19852 (19.5%) |  |
| **Packs smoked per day* years** | 17.8±26.7 | 17.7±26.6 | 1164 (1.14%) |
| **Drinking status** |  |  | 2881 (2.83%) |
| Never | 10116 (10.2%) | 10116 (9.9%) |  |
| Current/ Former | 88758 (89.8%) | 91639 (90.1%) |  |
| **History of hypertension** |  |  | 513 (0.50%) |
| No | 68194 (67.4%) | 68707 (67.5%) |  |
| Yes | 33048 (32.6%) | 33048 (32.5%) |  |
| **History of diabetes** |  |  | 538 (0.53%) |
| No | 94411 (93.3%) | 94949 (93.3%) |  |
| Yes | 6806 (6.7%) | 6806 (6.7%) |  |
| **Family history of lung cancer** |  |  | 781 (0.77%) |
| No | 87957 (87.1%) | 88738 (87.2%) |  |
| Yes/ Possible | 13017 (12.9%) | 13017 (12.8%) |  |
| **Physical activity level (min/week)** | 125.0±123.6 | 122.9±108.8 | 26059 (25.6%) |

**^a^** Values are mean (standard deviation) or counts (percentage) as indicated.
