# Supplementary material for: Higher carbohydrate quality, but not quantity, is associated with lower lung cancer risk in a population-based prospective cohort study
Source: Front Nutr. 2026 Jul 23;13:1857838. doi: 10.3389/fnut.2026.1857838 (PMC13441921; doi:10.3389/fnut.2026.1857838)
Supplement: Supplementary file 3 [file Table_3.DOCX]

| **Characteristics** | **Overall** | **Quartiles of low-carbohydrate diet score (LCD)** | | | |
| --- | --- | --- | --- | --- | --- |
|  |  | **Quartile 1 (≤10)** | **Quartile 2 (11-15)** | **Quartile 3 (16-20)** | **Quartile 4 (≥21)** |
| **Number of participants** | 101755 | 28881 | 24757 | 23776 | 24341 |
| **Low-carbohydrate Diet Score (LCD)** | 15.0±7.1 | 6.4±3.0 | 13.0±1.4 | 18.0±1.4 | 24.3±2.6 |
| **Age (years)** | 65.5±5.7 | 66.4±5.9 | 65.9±5.8 | 65.4±5.6 | 64.3±5.4 |
| **Gender** |  |  |  |  |  |
| Male | 49496 (48.6%) | 12464 (43.2%) | 11493 (46.4%) | 11947 (50.2%) | 13592 (55.8%) |
| Female | 52259 (51.4%) | 16417 (56.8%) | 13264 (53.6%) | 11829 (49.8%) | 10749 (44.2%) |
| **Race/ Ethnicity** |  |  |  |  |  |
| White | 94066 (92.4%) | 25368 (87.8%) | 23187 (93.7%) | 22446 (94.4%) | 23065 (94.8%) |
| Non-white | 7689 (7.6%) | 3513 (12.2%) | 1570 (6.3%) | 1330 (5.6%) | 1276 (5.2%) |
| **Education level** |  |  |  |  |  |
| Some college or less | 64953 (63.8%) | 18298 (63.4%) | 15499 (62.6%) | 15175 (63.8%) | 15981 (65.7%) |
| College graduate | 17848 (17.5%) | 4853 (16.8%) | 4493 (18.1%) | 4280 (18.0%) | 4222 (17.3%) |
| Postgraduate | 18954 (18.6%) | 5730 (19.8%) | 4765 (19.2%) | 4321 (18.2%) | 4138 (17.0%) |
| **Body mass index (kg/m^2^)** | 27.2±4.8 | 26.3±4.5 | 27.0±4.6 | 27.4±4.7 | 28.4±5.0 |
| **Smoking status** |  |  |  |  |  |
| Never | 48580 (47.7%) | 15544 (53.8%) | 12225 (49.4%) | 10899 (45.8%) | 9912 (40.7%) |
| Current/ Former | 53175 (52.3%) | 13337 (46.2%) | 12532 (50.6%) | 12877 (54.2%) | 14429 (59.3%) |
| **Cigarettes smoked per day when smoked** |  |  |  |  |  |
| Never-smoker | 48580 (47.7%) | 15544 (53.8%) | 12225 (49.4%) | 10899 (45.8%) | 9912 (40.7%) |
| 1-10 | 13596 (13.4%) | 4076 (14.1%) | 3390 (13.7%) | 3128 (13.2%) | 3002 (12.3%) |
| 11-20 | 19727 (19.4%) | 4992 (17.3%) | 4643 (18.8%) | 4856 (20.4%) | 5236 (21.5%) |
| ≥21 | 19852 (19.5%) | 4269 (14.8%) | 4499 (18.2%) | 4893 (20.6%) | 6191 (25.4%) |
| **Packs smoked per day* years** | 17.7±26.6 | 14.0±23.8 | 16.3±25.3 | 18.6±27.0 | 22.3±29.6 |
| **Drinking status** |  |  |  |  |  |
| Never | 10116 (9.9%) | 3792 (13.1%) | 2468 (10.0%) | 2008 (8.4%) | 1848 (7.6%) |
| Current/ Former | 91639 (90.1%) | 25089 (86.9%) | 22289 (90.0%) | 21768 (91.6%) | 22493 (92.4%) |
| **History of hypertension** |  |  |  |  |  |
| No | 68707 (67.5%) | 19620 (67.9%) | 16856 (68.1%) | 16111 (67.8%) | 16120 (66.2%) |
| Yes | 33048 (32.5%) | 9261 (32.1%) | 7901 (31.9%) | 7665 (32.2%) | 8221 (33.8%) |
| **History of diabetes** |  |  |  |  |  |
| No | 94949 (93.3%) | 27519 (95.3%) | 23306 (94.1%) | 22163 (93.2%) | 21961 (90.2%) |
| Yes | 6806 (6.7%) | 1362 (4.7%) | 1451 (5.9%) | 1613 (6.8%) | 2380 (9.8%) |
| **Family history of lung cancer** |  |  |  |  |  |
| No | 88738 (87.2%) | 25249 (87.4%) | 21704 (87.7%) | 20703 (87.1%) | 21082 (86.6%) |
| Yes/ Possible | 13017 (12.8%) | 3632 (12.6%) | 3053 (12.3%) | 3073 (12.9%) | 3259 (13.4%) |
| **Physical activity level (min/week)** | 122.9±108.8 | 133.1±113.8 | 124.4±107.8 | 119.2±107.0 | 112.7±104.3 |
| **Dietary factors** |  |  |  |  |  |
| Energy intake from diet (kcal/day) | 1738.6±736.4 | 1580.1±701.5 | 1683.0±673.6 | 1803.1±721.6 | 1920.3±801.9 |
| Carbohydrate intake (% E) **^b^** | 52.0±9.4 | 61.6±7.7 | 53.6±5.6 | 48.6±4.6 | 42.1±5.6 |
| Protein intake (% E) **^b^** | 15.4±3.0 | 13.5±2.4 | 15.2±2.7 | 15.7±2.6 | 17.6±2.6 |
| Fat intake (% E) **^b^** | 31.7±7.6 | 24.4±5.3 | 29.9±4.7 | 34.5±4.9 | 39.5±5.2 |

**Table S3.** Baseline characteristics of study population according to quartiles of low-carbohydrate diet score (LCD) **^a^**.

**^a^** Values are means (standard deviation) for continuous variables and percentages for categorical variables.

**^b^** %E the percentage of total energy intake.
