# Supplementary material for: Higher carbohydrate quality, but not quantity, is associated with lower lung cancer risk in a population-based prospective cohort study
Source: Front Nutr. 2026 Jul 23;13:1857838. doi: 10.3389/fnut.2026.1857838 (PMC13441921; doi:10.3389/fnut.2026.1857838)
Supplement: Supplementary file 4 [file Table_4.DOCX]

| **Smoking status** | **CQI Quartile** | **No. of participants** | **No. of cases** | **Person-years** | **Incidence rate per 1,000 person-years (95% CI)** |
| --- | --- | --- | --- | --- | --- |
| **Never-smokers** | Quartile 1 (≤10) | 14376 | 41 | 128967.4 | 0.32 |
|  | Quartile 2 (11-12) | 11271 | 36 | 101260.5 | 0.36 |
|  | Quartile 3 (13-14) | 11119 | 25 | 100052.6 | 0.25 |
|  | Quartile 4 (≥15) | 11814 | 39 | 105954.8 | 0.37 |
| **Current/former smokers** | Quartile 1 (≤10) | 18060 | 590 | 156036.5 | 3.78 |
|  | Quartile 2 (11-12) | 12350 | 347 | 107273.7 | 3.23 |
|  | Quartile 3 (13-14) | 11533 | 328 | 100420.5 | 3.27 |
|  | Quartile 4 (≥15) | 11232 | 300 | 97843.5 | 3.07 |

**Table S4.** Lung cancer incidence rates according to smoking status and quartiles of carbohydrate quality index (CQI).

The 95% confidence intervals(95% CI) for incidence rates were estimated using the exact Poisson method.
